# Selenium Toxicity in Plants and Environment: Biogeochemistry and Remediation Possibilities

**DOI:** 10.3390/plants9121711

**Published:** 2020-12-04

**Authors:** Mirza Hasanuzzaman, M. H. M. Borhannuddin Bhuyan, Ali Raza, Barbara Hawrylak-Nowak, Renata Matraszek-Gawron, Kamrun Nahar, Masayuki Fujita

**Affiliations:** 1 Department of Agronomy, Faculty of Agriculture, Sher-e-Bangla Agricultural University, Dhaka 1207, Bangladesh; 2 Citrus Research Station, Bangladesh Agricultural Research Institute, Jaintapur, Sylhet 3156, Bangladesh; razon_sau@yahoo.com; 3 Key Lab of Biology and Genetic Improvement of Oil Crops, Oil Crops Research Institute, Chinese Academy of Agricultural Sciences (CAAS), Wuhan 430062, China; alirazamughal143@gmail.com; 4 Department of Botany and Plant Physiology, University of Life Sciences in Lublin, Akademicka 15, 20-950 Lublin, Poland; bhawrylak@yahoo.com (B.H.-N.); renata.matraszek@up.lublin.pl (R.M.-G.); 5 Department of Agricultural Botany, Faculty of Agriculture, Sher-e-Bangla Agricultural University, Dhaka 1207, Bangladesh; knahar84@yahoo.com; 6 Laboratory of Plant Stress Responses, Department of Applied Biological Science, Faculty of Agriculture, Kagawa University, 2393 Ikenobe, Miki-cho, Kita-gun, Kagawa 761-0795, Japan

**Keywords:** abiotic stress, environmental pollution, oxidative stress, phytoremediation, Se bioavailability, trace element

## Abstract

Selenium (Se) is a widely distributed trace element with dual (beneficial or toxic) effects for humans, animals, and plants. The availability of Se in the soil is reliant on the structure of the parental material and the procedures succeeding to soil formation. Anthropogenic activities affect the content of Se in the environment. Although plants are the core source of Se in animal and human diet, the role of Se in plants is still debatable. A low concentration of Se can be beneficial for plant growth, development, and ecophysiology both under optimum and unfavorable environmental conditions. However, excess Se results in toxic effects, especially in Se sensitive plants, due to changing structure and function of proteins and induce oxidative/nitrosative stress, which disrupts several metabolic processes. Contrary, Se hyperaccumulators absorb and tolerate exceedingly large amounts of Se, could be potentially used to remediate, i.e., remove, transfer, stabilize, and/or detoxify Se-contaminants in the soil and groundwater. Thereby, Se-hyperaccumulators can play a dynamic role in overcoming global problem Se-inadequacy and toxicity. However, the knowledge of Se uptake and metabolism is essential for the effective phytoremediation to remove this element. Moreover, selecting the most efficient species accumulating Se is crucial for successful phytoremediation of a particular Se-contaminated area. This review emphasizes Se toxicity in plants and the environment with regards to Se biogeochemistry and phytoremediation aspects. This review follows a critical approach and stimulates thought for future research avenues.

## 1. Introduction

Selenium (Se) is a trace element present in small amounts found in almost all organisms. It is recognized both as beneficial and toxic, wherein the boundary between these two dual effects is very narrow and differs among plant species. The term selenium is derived from the Greek word “Selene”, which means moon [1,2]. Selenium is a member of the oxygen family (group 16), also called the chalcogens. The physiochemical characteristics of Se and sulfur (S) are very close, which results in non-specific binding of Se rather than S. These substitutions can disrupt the cell metabolism and alter the protein structures, causing toxicity [2]. Selenium distribution varies greatly throughout the globe [3]. In nature, Se hardly exists in elemental form and is found only in a few minerals [1]. The physical, chemical and biological mechanisms are responsible for the speciation of Se, which is determined mainly by the pH and redox state of the environment [4,5]. The uptake and metabolism of Se are also greatly varied in different soil and plant systems due to the heterogeneity of physiological and biochemical nature [6,7,8]. Naturally, it exists in copper (Cu) crystals, sulfides of Cu, lead (Pb), and gold (Au). Selenium is also a derivation of metallurgical engineering and becomes one of the significant environmental pollutants [2,9,10].

The importance of Se was described by Schwarz and Foltz [11] as an essential trace element in animal nutrition. They found the inclusion of Se in fodder crops blocked degenerative muscular disorder and chronic liver damage in mouse [2,11]. Later Reeves and Hoffman [12] suggested that the deficiency of Se in the human diet is the main reason for growth retardation, impaired bone metabolism, and abnormalities in thyroid function. Notably, certain areas of the world (e.g., Italy, Egypt, Turkey, Nepal) are Se-inadequate, whereas some are Se-toxic because of natural and anthropogenic events [13,14]. Thus, both Se-inadequacy and Se-toxicity are harmful to humans and animals [10]. According to the World Health Organization (WHO), Se dose in the human diet should be 50–55 μg day^−1^ [15,16,17]. In humans, Se-deficiency arises when its dietary intake is lower than 40 μg day^−1^, and chronic poisonous when the consumption exceeds 400 μg day^−1^ [18]. In livestock, the necessity of Se is 0.05–0.10 mg kg^−1^ dry forage, whereas the toxic dose in animal feed is 2–5 mg kg^−1^ dry forage [17]. Crops are one of the primary sources of Se for most of the organisms; therefore, Se-abundant crops could prevent Se-inadequacy [19,20]. 

It has been reported that Se supplementation in a relatively low amount can alleviate the negative impact of a variety of abiotic stresses in plants by improving growth and development [21]. For example, Se application via plant roots restricted the uptake and translocation of heavy metals/metalloids [22,23,24]. On the other hand, Se toxicity, impeding plant growth, development, and disturbing plant ecophysiology, causing chlorosis and necrosis, restricted growth, and reduced protein biosynthesis [25,26,27]. Selenium toxicity or selenosis can occur in two ways, i.e., forming seleno-proteins and inducing oxidative stress. For instance, Se concentration ≥2 mg kg^−1^ dry weight (DW) in *Arabidopsis*, is toxic and cause a 10% reduction in the biomass without visible symptoms [28]. Selenium toxicity is reliant on the age of the plant and the chemical form of this element. The lowest concentration of Se, causing a significant decrease in the biomass of cucumber (*Cucumis sativus*) was 20 µM for SeO_3_^2–^ and 80 µM for SeO_4_^2–^ [29]. Similarly, SeO_3_^2–^ (50 or 100 μM) reduced vegetative growth and disrupted reproductive development [30]. Moreover, Se (SeO_3_^2–^, 0.1 and 0.5 µM) decreased δ-aminolevulinic acid (ALA) content in maize (*Zea mays*) [31]. Selenium was also found to interact with other toxic metal/metalloid and accelerate the toxic effects [32]. 

Many plants efficiently take up Se and could be implemented to remove Se from the contaminated areas by several phytoremediation approaches such as phytoextraction, phytovolatilization, and rhizofiltration [33,34]. For instance, owing to the high level of accumulation, *Brassica napus* and *B. juncea* have been used for the Se phytoextraction [35]. For Se volatilization, *Astragalus bisulcatus* (Se hyperaccumulator) were used to volatilize the Se from the contaminated environment [36]. Notably, among numerous plant species, *B. oleracea* and *A. bisulcatus* volatilize higher Se, afterward *Medicago sativa* and *Solanum lycopersicum* [37]. For Se rhizofiltration, *Typha angustifolia* grown in wetland environments has been described in the elimination of supplemented Se as SeO_3_^2–^ (89%) and SeO_4_^2–^ (46%) [38], whereas muskgrass (*Chara canescens* Desv. and Lois) eliminate approximately 70–75% Se from the aqueous condition [39]. In recent decades, a great progress has been made using genetically engineered plants to remove metals [40]. Although there has been much interest in the dual role of Se in plants, the detail mechanism of Se toxicity and its remediation has not been summarized. 

Therefore, in this review, we have discussed the recent advances in the Se toxicity in the plants and environment in the light of the recent research endeavors and experimental evidence. Moreover, Se biogeochemistry and phytoremediation possibilities have also been summarized, which would stimulate thought for future research avenues.

## 2. Selenium Biogeochemistry

**Figure 1:**
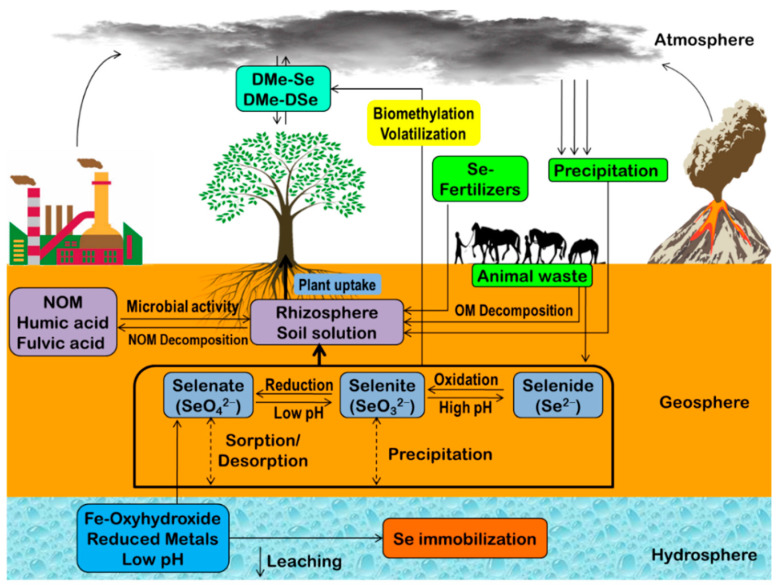
The schematic diagram for the mechanisms of Se-biogeochemistry in different Earth’s compartments. DMe-Se—dimethyl selenide; DMe-DSe—dimethyl diselenide; NOM—natural organic matter, OM—organic matter.

After the discovery in 1817 by Swedish chemist J.J. Berzelius, Se was considered a toxic element. Meanwhile, Se was found in all four compartments of the Earth, i.e., viz. atmosphere, hydrosphere, geosphere, and biosphere, ranked as the 67th abundant element on Earth (Figure 1) [41]. Moreover, it was placed 145th among the toxic and hazardous ingredients and 125th as the priority pollutant. Chemically Se resembles S and is coupled with the S depositions in the geosphere, such as coal [42]. The content of Se in coal ranged from 1–43 mg kg^−1^. The principal sources of terrestrial Se are Se rich minerals. Elemental Se or Se ore are very rare; nevertheless, Se can be combined with other elements, e.g., nickel (Ni), S, Cu, Ag, and Pb. Among the anthropogenic sources, industrial activities (pharmaceuticals production, ceramics factories, glass industry) mainly release Se [41]. 

In Earth’s crust, sedimentary rocks contain a higher amount of Se than magmatic rocks. In contrast, organic-rich soils contain Se up to 600 mg kg^−1^ of soils and could adsorb more from water by silt and clay particles [43]. Naturally occurring 407 Se minerals are known, of which 259 are unique and divided into several classes (class I (elemental Se), class II (“sulfides”, includes 182 selenides), class IV (“oxides”, includes 49 selenites), and class VI (“sulfates”, includes four selenates)). Moreover, these Se containing minerals are classified according to their valences (−II, −I, 0, +IV, and +VI), for instance, metal selenide (Se^–^, Se^2–^), elemental Se (Se^0^), thioselenate (SSeO_3_^2–^), selenite (SeO_3_^2–^), selenate (SeO_4_^2–^), respectively. Among the phases of Se, only one is recognized as organic, but yet not identified completely [44]. Hydrocarbons are also an important source of Se, but still, many remained undiscovered. In nature, physical (sorption effects of soils and sediments), chemical (pH, redox potential, organic matter content, and competitive ions), and biological (reduction, alkylation, dealkylation, and oxidation of Se by bacterial strains) mechanisms determined Se biogeochemistry (reactivity, mobility, and bioavailability) and are responsible for the speciation of this element [4,5].

### 2.1. Chemical Mechanisms Regulating Se Biogeochemistry

The availability of Se increases at higher pH. Environmental pH (soil or water) plays a vital role in the speciation of inorganic Se compounds. Selenium forms can remain oxidized or as diprotic acids (selenous and selenic acids) to maintain an acid-base equilibrium [44]. Under normal water pH (5.0–9.0), the dominant Se forms are HSeO_3_^−^ and SeO_3_^2−^, which remain in an equal fraction at pH 8.54 [45]. Further pH increases fully deprotonated, producing SeO_4_^2−^ (approximately 100%). The inorganic Se compounds are predominantly present in the anionic form (SeO_4_^2−^, HSeO_3_^−^, and SeO_3_^2−^), therefore, the bioavailability of Se will decrease with the decreasing pH [46]. In contrast, in strong acidic pH, Se-containing minerals dissolutes and releases Se; as a result, its mobility increases. Besides, adsorption by Fe-oxyhydroxides and co-precipitation with calcite abolishes the mobility of Se, thus abates Se-leaching under alkaline pH [47].

Redox potentially influences Se speciation too. Strong oxidizing conditions facilitate SeO_4_^2−^ formation, while mild oxidizing conditions enhance HSeO_3_^−^ formation [46]. In contrast, elemental Se and volatile H_2_Se are predominant in strong reducing conditions. At strong reducing conditions and pH ≥ 4.0, HSe^−^ shows dominancy while elemental Se remains insoluble and Se^2−^ binds with metals [48]. 

Humic and fulvic acids of variable molecular weight contained by natural organic matter (NOM) bind Se to their functional groups containing nitrogen (N), S, and/or oxygen (O) [49]. Some reports suggest that Se concentration is positively correlated with NOM content. Interestingly, Se sorption by NOM is dependent on the NOM solubility, which is also ionic strength and pH-dependent [44]. Humic substances are also highly associated with Se, and upon adding SeO_3_^2−^ to the forest soil, Se was readily incorporated into the humic substances. 

Competitive ions can reduce the availability of Se. Generally, an increase in pH was found to increase the sorption of metal cations on humic acid and immobilize SeO_3_^2−^. Additionally, at low pH, the presence of Fe-oxyhydroxide immobilizes SeO_3_^2−^ [44]. Moreover, humic acids with redox potential from 0.4 to 0.8 V can reduce Cu(0), Cu(I), Sn(II), and U(IV) oxides, as well as Hg(II) and Fe(III), forming metal ion complexes and decrease the availability of Se [50].

### 2.2. Physical Mechanisms Regulating Se Biogeochemistry

Selenium bioavailability is affected by the mineral compositions of soils and sediments. Soil pH and the redox potential determine Se sorption on the aluminum (Al), iron (Fe), and manganese (Mn) oxyhydroxides. Only on the calcareous and the montmorillonite soils Se sorption was not affected at pH 2.0–9.0 along with 27 to 270 mg L^−1^ of SeO_4_^2−^. Moreover, on the goethitic soils, SeO_3_^2−^ have weaker sorption, influenced by the pH and the SeO_4_^2−^ concentration. For example, a high SeO_4_^2−^ (300 mg L^−1^) concentration shows strong sorption at acidic pH, while lower near neutral. However, if the initial concentration of SeO_4_^2−^ is ≤30 mg L^−1^ no sorption was shown at pH 2.0–9.0. Notably, goethitic soils adsorbed SeO_4_^2−^ by a weak electrostatic attraction, which facilitates the plants to uptake Se easily. On the contrary, Fe and Al oxides of soils and sediments strongly adsorb SeO_3_^2−^, where maximum sorption was observed at pH 3.0–4.0 and declined thereafter up to pH 8.0 [45,51].

Abiotic reactions could contribute to Se redox speciation, induced by sulfides, thiols, and ascorbic acid (AsA). For example, cysteine (Cys), can potentially reduce SeO_3_^2−^ to Se, which increases from pH 5.0–7.0, and decrease thereafter up to pH 9.0. On the contrary, cations (Mg^2+^ and Ca^2+^) can decrease the rate of Cys-induced SeO_3_^2−^ reduction by forming Cys-cations complexes [52]. Moreover, the bioavailability of sulfides influenced Se availability reversely. Similarly, the transformation of SeO_3_^2−^ to Se^0^ is influenced by the AsA level, which decreases from pH 2.0–5.5 affecting Se uptake by the plants. Moreover, temperature also affects the activation energy to reduce SeO_3_^2−^ to Se^0^ [52].

### 2.3. Biological Mechanisms Regulating Se Biogeochemistry

Microorganism-induced catalysis regulating Se speciation is the key biological mechanism for Se biogeochemistry, which affects the mobility and the bioavailability of Se [53,54]. Generally, assimilatory and disassimilatory reduction, alkylation, dealkylation, and oxidation are the primary transformation mechanisms for speciation of Se. Microbial reduction of SeO_4_^2−^ and SeO_3_^2−^ to Se is often used for bioremediation. Afterward, Se^0^ could further be reduced to Se^−^/Se^2−^, which is stable under reducing condition. However, these Se^−^/Se^2−^ may react with metals (zinc, Zn and cadmium, Cd), forming highly insoluble metal-Se^−^/Se^2−^ and reduce Se availability [55]. Moreover, alkylation leads to volatile dimethyl selenide (DMe-Se) and dimethyl diselenide (Dme–DSe) formation [56].

## 3. Selenium in the Environment

**Figure 2:**
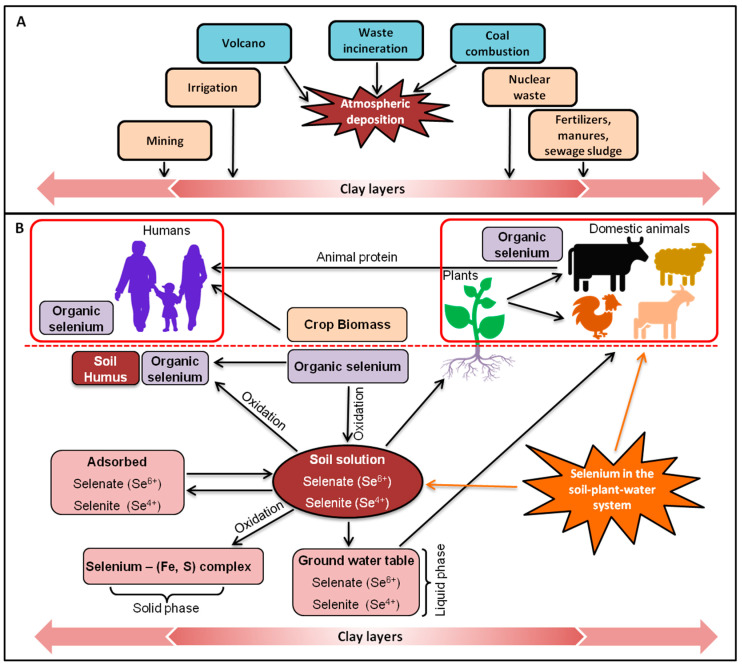
An overview of Se in the environment. (**A**) Sources of Se in the environment; (**B**) Selenium in the soil-plant-water consumer system. Plant roots take up Se (selenate or selenite) from the soil solution. The Se concentration in the soil solution depends on the solubility of the Se forms occurring and the biological transformations of organic forms. Modified from Hasanuzzaman et al. [59].

Selenium occurs in different environmental compartments in different forms (Figure 2). In nature, both organic (gaseous (DMe-Se, C_2_H_6_Se; DMe-DSe, C_2_H_6_Se_2_) and nongaseous (selenocysteine, Se-Cys; selenomethionine, Se-Met; Se-methylselenocysteine, SeMe-SeCys)) and inorganic (Se^0^, Se^−^, Se^2−^, SeO_3_^2−^, and SeO_4_^2−^) forms are found. On the other hand, the speciation of Se determines its accessibility and dispersal based on many features, like pH, NOM, microbial activity, redox potential, ionic elements, soil quality, temperature, and moisture [57,58].

Naturally, Se exists in stratified rocks established during the Paleozoic era to the Cenozoic era [60]. The two anionic inorganic forms of Se (SeO_3_^2–^ and SeO_4_^2–^) are readily soluble, moveable, bioavailable, and highly toxic. Organic Se originates mainly from the decaying of plants accumulating Se [60,61]. Globally, the mean Se level in soils varies from 0.1–0.7 mg kg^−1^, whereas clay soils contain 0.8–2 mg kg^−1^ and tropical soils 2–4.5 mg kg^−1^. The amount of Se in the soil depends on its texture, level of organic matter, and rainfall [62]. Notably, clay soils have higher Se content than coarse soils [63]. Volcanic soils and igneous rocks have a very low content of Se, found in mountainous countries like Finland, Scotland, and Sweden. Contrary sedimentary rocks are enriched with Se and incline to be mobile in rocks of the arid part climate, where it exerts harmful impacts on livestock [64,65].

Selenium content in plant originated food varies due to soil Se level, which depends on the ability of plants to accumulate Se, as well as the geomorphological area [2,9]. Se accumulation level differs remarkably, from 0.006 to 3.06 μg g^−1^ DW in inadequate regions (few areas of New Zealand, Sweden, and Canada; [9]). Usually, fruits accumulate lower amounts of Se than vegetables. Similarly, cereal crops accumulate Se in their seeds, particularly in the form of Se-Met and Se content varies from 0.01–0.55 μg g^−1^. Besides, Grasses normally have a high amount of Se compared to legumes, and cow milk and other farm products contain 0.001–0.17 μg Se g^−1^ fresh weight (FW) [66,67]. Additionally, Brazil nuts, *Brassica* spp. (cabbage, broccoli, and mustard, etc.), garlic, and *Astragalus* spp. accumulate a great amount of Se and a good source of Se in the diet [68,69,70,71].

Selenium enters in groundwater from sediments, soil wastes and sub-soils, containing Se. Moreover, Se level in groundwater increases due to excessive use of Se-enriched fertilizers for instance; Western European countries like Belgium and France (0.12 μg L^−1^ and 2.4–40 μg L^−1^, respectively, and some Se-enriched west regions of Punjab, India (341 μg L^−1^) [9,18,72]. Therefore, Se concentration in drinking water should not exceed 10 μg L^−1^, together optimum human intake ought not to exceed from 55–200 μg day^−1^ for adults [73]. Elsewhere, Se level in seawater varies from 4000 to 12,000 μg L^−1^ [18].

Environmental pollution by human activities (burning of papers, tires, and fossil fuels etc.) and natural phenomena (wildfire and soil erosion) also introduces Se into the atmosphere [18]. In the atmosphere, Se usually exists as volatile organic compounds, such as diethylmaleate-selenide (DEMSe), DMe-Se, DMe-DSe, methaneselenol (CH_3_Se), and inorganic selenium dioxide (SeO_2_). Besides, SeO_2_ is volatile and transformed to selenious acid (H_2_SeO_3_). The level of the Se in the atmosphere varies between 1–10 ng m^−3^ and particularly lower than water and soil [9].

## 4. Selenium Abundance: A Global Distribution

The relationship between Se level in various soil/plant types and human health have been widely studied [58,62,74,75,76,77,78,79]. Regarding humans, it has been described that the Se uptake by an individual and the Se status in the population is closely related to Se level in the soil and the edible plants cultivated in that particular area.

**Figure 3:**
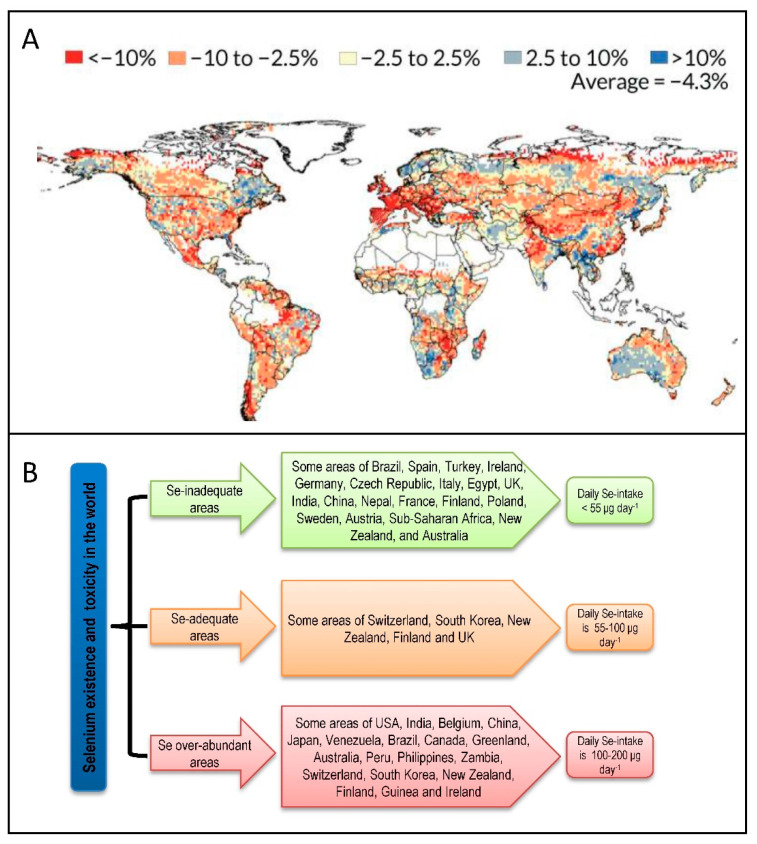
(**A**) Predicted percent changes in soil Se concentration from 1980–1999, 2080–2099. (**B**) Overview of Se occurrence and toxicity in various areas of the world based on human population and soil Se-availability. Source: LSA Honors Physics [89]; Hasanuzzaman et al. [21] (with permission from Elsevier).

Selenium toxicity has been reported in several regions around the world, including west-central San Joaquin Valley of California, Colorado, Utah, Wyoming, and Idaho, US [80,81,82], Enshi district of Hubei province, China [83,84], Canada [85], Australia and New Zealand [73,86], West Bengal areas and northwestern Punjab regions, India [87,88]. On the other hand, in different regions, Se level is predicted to be decreased in the coming decades (Figure 3A).

Besides, many regions around the world are Se-inadequate. Countries such as France, Belgium, Brazil, Serbia, Slovenia, Spain, Portugal, Turkey, Poland, Germany, Denmark, United Kingdom, Slovakia, Austria, Ireland, Greece, Argentina, Italy, China, Nepal, Saudi Arabia, India, Czech Republic, Croatia, Egypt, Uruguay, Burundi, and New Guinea are accounted to have Se-inadequate areas (Figure 3B) [13,90,91,92,93]. Similarly, many regions are rich in Se, e.g., northwestern Punjab areas, India; Enshi district of Hubei, China; mountain and coastal communities, Japan; the Orinoco, Venezuela; western parts; Pakistan; western parts, Canada; Greenland; and Australia [72,83,86,94,95,96,97,98,99,100]. Almost 80% of the world’s total Se stocks are found in Canada, United States, Belgium, China, Peru, Chile, New Guinea, Zambia, Philippines, Zaire, and Australia [101].

Selenium malnutrition is a rising problem for humans globally, including Europe, Africa, Asia (mainly China), Australia, New Zealand, and southern US states. Almost forty countries documented having Se-inadequate regions, which is related to inadequate Se uptake (10 μg day^−1^) or even low in human, Clinical experimental reports suggested that good health is correlated with proper daily Se uptake, which can influence billions of people around the world. Selenium-deficiency can cause muscle weakness, pain/swelling or redness of joints and muscles, cancer susceptibility, erythrocytes fragility, unusual skin complexion, cardiomyopathy, Keshan, and Keshin-Beck diseases (KBD). For instance, 72% of the total area of China are Se-inadequate, where peoples are at risk of Keshan diseases, which is related to cardiomyopathy [102,103]. These problems can be solved by the development of Se-enriched foods (plants and products). On the other hand, animal health is also negatively influenced by Se-inadequacy; therefore, plant-based Se-supplementation may solve this problem for producing Se-enriched animal products [93]. However, the sufficient (0.05–0.10 mg kg^−1^) and toxic (4 to 5 mg kg^−1^) Se dose in the animal diet should be considered before administration [104]. For example, China has some highly Se-enriched soils, where they grow arable crops confirming sufficient regular Se intake by animals and humans [105,106].

On the other hand, Se toxicity has adverse effects in various regions around the globe. Meanwhile, Se toxicity involves kidney and liver failure, necrosis of heart and liver, nausea or vomiting, blood coagulation, loss of hair and nails, memory loss and tingling [28,107,108,109]. Therefore, it should be conscious regarding Se-concentration in food and drinking water.

## 5. Selenium Toxicity in Plants

### 5.1. Toxic Effects on Plant Growth and Development

**Figure 4:**
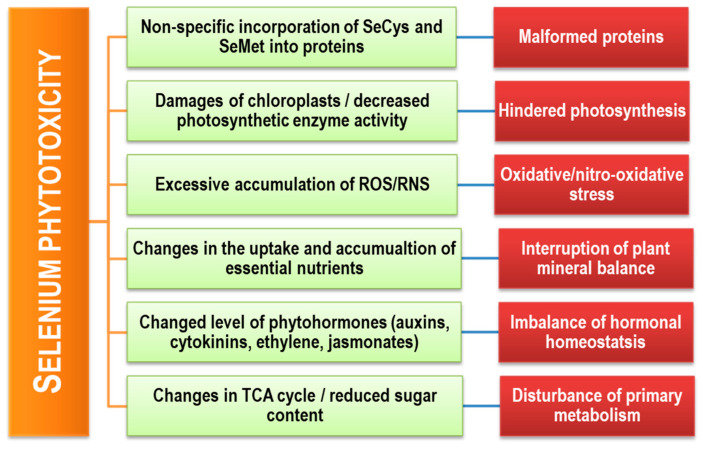
A schematic representation of the causes and consequences of Se toxicity in plants.

Selenium toxicity depends on the plant species, its age, and the availability of Se (Table 1; Figure 4) [30]. Young plants are much more sensitive to Se-toxicity than mature ones, and SeO_3_^2–^ is more phytotoxic than SeO_4_^2–^. Selenate is as toxic as SeO_3_^2–^ only in the case of green algae *Chlamydomonas reinhardtii* (Figure 4) [110,111]. However, it has been suggested that Se is an essential element in this algal species because of the Se-dependent GSH peroxidase (GPX) component [112]. In the plant, higher toxicity of SeO_3_^2–^ than SeO_4_^2–^ is because of the faster incorporation of SeO_3_^2–^ into the Se-amino acids in roots after uptaken [113]. Previously, Hopper and Parker [114] reported that SeO_3_^2–^ was more toxic than SeO_4_^2–^ in perennial ryegrass and strawberry clover, and SeO_3_^2–^ preferentially inhibited the root growth, whereas SeO_4_^2–^ impeded shoot growth. In a recent study, Moreno et al. [115] reported that the maximum SeO_3_^2–^ concentration without growth inhibition for *Raphanus sativus*, *Helianthus annuus*, *Medicago sativa*, and *Beta vulgaris* to be 1, 10, 0.25, and 0.25 mg L^−1^, respectively. Notably, the uptake of SeO_3_^2–^ by roots and subsequent distribution within plants is slower than SeO_4_^2–^ [116].

The toxicity thresholds for SeO_3_^2–^ and SeO_4_^2–^ in lettuce and cucumber were compared [29,119], where SeO_3_^2–^ was more toxic than SeO_4_^2–^ but both Se forms efficiently reduced roots biomass than shoots. In this context, a specific key role of the SULTR1;2 transporters regarding SeO_4_^2–^ sensitivity was found in *Arabidopsis* [132], which suggested that root growth, particularly root tip activity, might be a specific target of SeO_4_^2–^ toxicity in plants [132]. Furthermore, phytotoxic Se (both SeO_3_^2–^ and SeO_4_^2–^) induced biomass reduction was accompanied by a corresponding decrease in leaf area [120]. Selenium supplemented irrigation water (10, 100, and 1000 µM SeO_3_^2–^) did not affect the height of coffee plants, but the DW of roots and leaves, as well as the leaf area, was reduced, but the leaf thickness was increased at the maximum Se concentration [133]. Similarly, barley plants treated with different Se concentrations (2–16 ppm SeO_4_^2–^) exhibited a reduction in plant height and chl content regardless of Se concentration, but the damaging effect was dose-dependent [126].

A positive correlation was found between the thickness, and specific root volume of lateral roots indicated the Se-inducted production of endogenous ethylene [121], which was suggested previously by Konze et al. [134]. They reported that two Se-amino acids, i.e., SeMet and selenoethionine, serve as ethylene precursors and enhanced ethylene production in auxin-treated *Pisum sativum* stem sections and senescing flower tissue of *Ipomea tricolor*. Moreover, Se toxicity induced cell viability inhibited root growth, and malformed root architecture, consequently reduced primary root elongation and facilitate lateral root growth [121]. In *Brassica juncea,* SeO_4_^2–^ and SeO_3_^2–^ (20 µM) treatment accumulated endogenous Se in floral parts and impaired pollen germination [124]. Moreover, Lehotai et al. [30] observed SeO_3_^2–^ (50 or 100 μM) reduced vegetative and inhibited reproductive development. In turn, SeO_4_^2–^ treatment (20 μM) altered hormonal balance, which alters morphological characteristics and modifies growth.

### 5.2. Toxic Effects on Physiological Processes

Selenium excess negatively affects several physiological and biochemical processes in plants. Among them, one of the foremost negative effects is the reduction of chl biosynthesis resulted in chlorosis. Saffaryazdi et al. [122] showed that SeO_3_^2–^ >1 mg L^−1^ in the nutrient solution decreased chl content in spinach, which might be related to lipoxygenase (LOX)-mediated lipid peroxidation and changes in the activity of antioxidant enzymes and/or negatively altered synthesis and activity of porphobilinogen synthetase (δ-aminolevulinate (ALA) dehydratase) required for chl biosynthesis [135]. Supporting this, Jain et al. [31] showed 0.1 and 0.5 µM SeO_3_^2–^ decreased ALA content in etiolated maize. Besides, Lehotai et al. [30] found an excess of Se diminished the chl *a*, chl *b*, chl *a*/*b*, total chl, and total carotenoid accumulation [30].

Selenite at concentrations >20 µM impaired the values of *Fv*/*Fm*, *Fo*, and *Fm* in hydroponically grown cucumber. However, SeO_4_^2–^ (2–80 µM) did not influence the chl fluorescence parameters [19]. A similar result was also postulated by Valkama et al. [136] using SeO_4_^2–^ (1 mg kg^−1^ soil) on. However, a high soil Se level increased sensitivity to enhanced UV-B radiation in strawberry. A high Se concentration increased quantum efficiency of PS II but decreased the number of leaves, biomass, and the starch/chloroplast area ratio, which was related to changes in the activity and/or biosynthesis of enzymes, rather than alteration of photosystem II (PSII [136]).

Both SeO_3_^2–^ and SeO_4_^2–^ (4.5 ± 0.2 µM) caused different toxic symptoms in *Chlamydomonas reinhardtii*, including ultrastructural damages of appressed domains of the chloroplast, subsequently interrupt photosynthetic electron chain, inhibit photosynthetic electron transport, and hindered photosynthesis [111]. Wheat exposed to 100 μM SeO_4_^2–^ showed decreased PSII and PSI system activities [120]. Moreover, accumulation of free amino acid selenocystathionine together with SeCys and SeO_4_^2–^ in SeO_4_^2–^ (20 μM) exposed *Stanleya albescens*, showed reduced growth, necrosis and chlorosis, and photosynthesis disorder [127]. In *A. thaliana*, Se-induced growth inhibition was attributed to decreased stomatal density and impaired stomatal regulation together with reduced cell viability [26].

Phytotoxic Se-induced growth reduction could be a consequence of disturbances in the balance of mineral nutrition. By modifying the uptake, accumulation, and transport of mineral nutrients, Se affects different biochemical reactions and physiological processes (growth, photosynthesis, respiration, gas exchange, water uptake, phloem unloading, and activation of protease inhibitor genes). In addition, Se could reduce or intensify the toxicity of essential or toxic elements by limiting or aggravating stresses induced by these elements. Kopsell et al. [137] observed decreased foliar concentrations of B, Fe, and P along with increased S and K in SeO_4_^2–^-treated *Brassica oleracea* L. Similarly, Madagascar periwinkle, treated with both SeO_3_^2–^ and SeO_4_^2–^, resulted in elevated Zn and Cu phytoaccumulation, and enhanced carbohydrate and alkaloids accumulation capacity, but this effect was more pronounced by SeO_3_^2–^ than SeO_4_^2–^ exposure [138].

Maize treated with SeO_3_^2–^ (5–100 µM) showed increased P and Ca but decreased K content [131]. However, Ca bioconcentration and an opposite P reduction was found in SeO_4_^2–^-treated tall fescue and white clover [139]. Beyond, a synergic effect of Se and Fe was also found under Se exposure, where Fe concentration increased with the growing tissue Se concentration. The effect of Se was tested in a separate study using lettuce, which showed increased shoot Se concentrations, but decreased macronutrient accumulation, N, P, K, Ca, Mg, and S in leaves of lettuce together with growth reduction symptoms [129]. Besides, Se in the nutrient solution (10 and 20 mg L^−1^ SeO_3_^2^) and to soil (50 and 100 mg kg^−1^ SeO_3_^2–^) reduced bioaccumulation of Mg, K, P, Fe, Cu, and Zn in *Pteris vittata* [128].

Selenite-exposed maize treated or untreated with auxin (IAA) exhibited lower Mg content in the leaves and roots in comparison to mesocotyls. Moreover, Se supplementation increased the shoot Fe content [140]. In cucumber, 80 µM SeO_4_^2–^ is recognized as toxic, markedly reduced shoot K content but elevated amounts of Ca and S-SO_4_. In turn, SeO_3_^2–^ (>20 µM) caused a severe decrease in the P, K, Mg, Ca, and S-SO_4_ levels in shoots [117].

Selenium toxicity could be coupled with metal toxicity. In wheat and pea, Se (SeO_3_^2–^ and SeO_4_^2–^) enhanced Cd and Cu uptake and toxicity and altered their distribution, especially in the SeO_3_^2–^-treated peas. Besides, in wheat shoots, SeO_4_^2–^ elevated Cd bioaccumulation up to 50%, while Cd bioaccumulation increased up to 300% in pea roots by SeO_3_^2–^ supply [141]. In *Brassica juncea*, the use of SeO_4_^2–^ (50 mg Se L^−1^) and iodide (100 mg L^−1^) showed synergistic actions in reducing nitrate accumulation, enhancing flavonoid biosynthesis, increasing B and Al accumulation, and decreasing Sr and Cd bioconcentrations [142].

It was reported that the change in the transport abilities of some nutrient ions is one of the first observed symptoms of Se effects on plants [143]. It was found that SeO_3_^2–^ (2–10 µM) in the rooting medium inhibited root elongation in wheat, which is further enhanced by CaCl_2_, MgCl_2_, SrCl_2_ supplementation, along with pH reduction [144]. Moreover, these compounds raised the plasma membrane activity, enhancing Se uptake by roots. Yet, Bailey et al. [145] observed SO_4_^2–^/SeO_4_^2–^ antagonism using SeO_4_^2–^ antagonist SO_4_^2–^ in wigeon grass. Similar SO_4_^2–^/SeO_4_^2–^ antagonism has also been confirmed by White et al. [146] in *Arabidopsis*. The results of these studies show that increased levels of SO_4_^2–^ in the growing media increased FW and enhanced S accumulation, but reduced the Se content in shoots. On the contrary, the increase in the SeO_4_^2–^ concentration in the growth media increase both Se and S content in shoots but reduced shoot FW. However, the SO_4_^2–^/SeO_4_^2–^ antagonism seems to be stronger than the PO_4_^2–^/SeO_3_^2–^ antagonism [114]. Since SeO_4_^2–^ is transported across the cell membrane by high-affinity SO_4_^2–^ transporters, there is strong evidence that SeO_4_^2–^ directly competes with SO_4_^2–^ for uptake by plants, whereas PO_4_^2–^ transporters are involved in the SeO_3_^2–^ transport [147,148]. Therefore, the application of SeO_3_^2−^ increased the foliar concentration of S in lettuce shoots. Following the uptake, SeO_3_^2–^ is easily transformed into organic Se in roots, while SeO_4_^2–^ is quickly translocated and either metabolized or stored in plastids using the S metabolic pathway [19,91,148]. Both SeO_4_^2–^ and SeO_3_^2–^ are assimilated as their S analogs by the same pathway, leading to Se incorporation in almost all S metabolites. For that reason, Se non-accumulator plants contain higher amounts of Se in proteins than Se-accumulators [21].

The physicochemical differences between Se and S result in small but very significant changes in the biological properties of Se-substituted proteins. Although the Se-Se bond is longer and weaker but is more labile than S–S bonding and alters tertiary protein structure leading to catalytic malfunction function of enzymes. Due to its higher nucleophilicity, SeCys is more reactive than Cys, and replacement of Cys by SeCys induce Se-Se bridges preventing the formation of S-S bridges, thus altering redox potential and enzyme kinetics [149].

The Fe-S cluster proteins of the chloroplastic and mitochondrial electron transport chain are very much prone to SeCys replacement [150]. Due to its larger size, the Fe–Se cluster does not fit properly in proproteins. Hallenbeck et al. [151] found that the replacement of the Fe–S cluster with Fe–Se extremely reduced the activity of *Klebsiella pneumoniae* nitrogenase. Conversely, such substitution was beneficial for the activity of *Citrus sinensis* glutathione (GSH)-dependent peroxidase expressed in *Escherichia coli* [152]. Due to the crucial role of Cys residues in the protein structure, SeCys substitution is more detrimental than SeMet substitution [149]. Replacement of Met with SeMet disturbs protein synthesis via impairing the formation of the peptide bond [153]. However, SeCys having easy deprotonation capacity is more reactive than Cys and impaired protein function [154]. Coffee leaves supplied with toxic SeO_3_^2–^ (10 µM) showed an increased content of caffeine and soluble sugars as well as decrease photosynthetic pigments. Substantially lower nitrate reductase (NR) activity and disturbances in the free amino acid profile were found as well [133]. Moreover, Zhang et al. [155] demonstrated a high concentration of SeO_3_^2–^ inhibited the growth of chickpea sprouts and the biosynthesis of formononetin and biochanin an isoflavones.

### 5.3. Selenium-Induced Oxidative Stress in Plants

**Figure 5:**
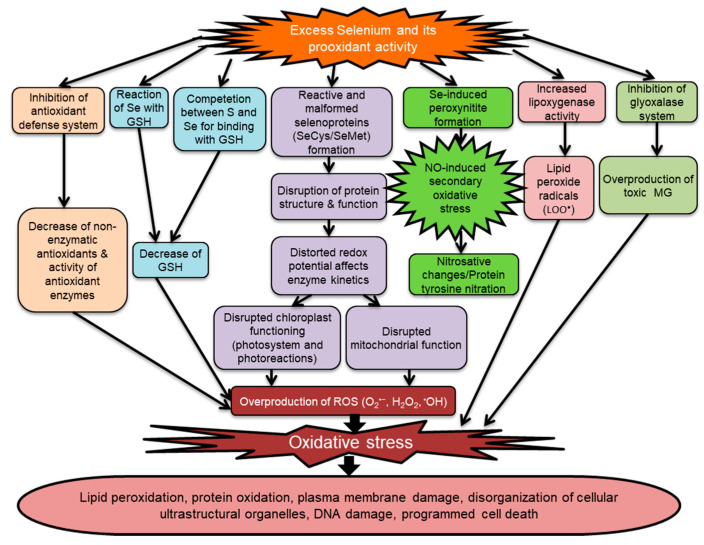
Selenium-induced oxidative stress and consequent damage to the plant cell. The inhibition of the antioxidant defense system by Se excess provokes overproduction of reactive oxygen species (ROS). Excess of Se can directly react with several metabolites to generate ROS. Moreover, modified chloroplast and mitochondrial reactions under toxic Se concentrations cause ROS overproduction. Selenium causes nitric oxide-induced secondary nitrooxidative stress. Increasing lipoxygenase (LOX) activity under Se exposure generates peroxide radicals (LOO^•^) and inhibits the glyoxalase system causing methylglyoxal (MG) toxicity and subsequent oxidative stress.

Being prooxidative, Se provokes oxidative stress. Different mechanisms have been described to explain Se-aggravated oxidative stress and its damaging effects in the plant cell (Figure 5; Table 2). Selenite restrains the ubiquitin-proteasome pathway and persuades the pentose phosphate pathway. Moreover, Se-induced inhibition of antioxidant defense provokes the overproduction of reactive oxygen species (ROS) [156]. Moreover, Se can directly react with several metabolites to generate ROS. The reaction of SeO_3_^2–^ with GSH causes O_2_^•−^ generation and subsequent H_2_O_2_ overaccumulation. Therefore, excess Se causes depletion of GSH level and its activity, encouraging ROS production.

Selenium toxicity also induces reactive and malformed selenoproteins (SeCys/SeMet), thus alter redox potential, which distorts chloroplastic and mitochondrial enzyme kinetics [159]. Besides, Se toxicity disrupted chloroplast ultrastructure and function (photosystem and photoreactions) as well as upsetting mitochondrial functioning, causing ROS overgeneration. Selenite-induced overproduction of mitochondrial O_2_^•−^ and consequently diminished aconitase activity activate the alternative oxidase pathway [150]. In turn, Se-induced peroxynitrite formation creates nitrosative stress. Therefore, nitrosative changes in protein (tyrosine nitration) occurs [30]. Yet, a Se-mediated increase in LOX activity generates lipid peroxide radicals (LOO^•^) [162]. The mechanisms accountable for Se-induced oxidative stress in plants are illustrated in Figure 5 and Table 2.

Both SeO_4_^2–^ and SeO_3_^2–^ induces ROS overaccumulation and oxidative stress in plant cells. In vitro investigations indicated that SeO_3_^2–^ reacts with GSH to produce O_2_^•–^ [163]. Accordingly, *Arabidopsis* mutant vtc1 with defective AsA biosynthesis accumulated more O_2_^•–^ and H_2_O_2_ under SeO_3_^2–^ exposure than wild-type plants, which indicated that the antioxidant properties of AsA reduce ROS accumulation in plants exposed to SeO_3_^2–^ [164]. Dimkovikj and Van Hoewyk [165] found SeO_3_^2–^-induced quick formation of mitochondrial O_2_^•−^ and consequent decrease in aconitase activity, which activated the alternative oxidase pathway. They also found an increased glucose concentration with higher respiratory rates and ATP levels. Selenite exposure also increases GSH concentrations along with elevated levels of γ-glutamyl cyclotransferase, which degrade Se metabolites conjugated to GSH.

At a high concentration, Se acts as a prooxidant. Hartikainen et al. [152] found an enhanced SOD activity and increased tocopherol content in ryegrass due to excess Se (>10 mg kg^−1^), which indicated the prooxidative activity of Se. They also observed excessive accumulation of toxic LOO^•^, which was scavenged by α-tocopherol to produce LOOH, and subsequently converted to less toxic LOH by enhanced GPX activity. Additionally, increased SOD activity prevents O_2_^•−^ accumulation. Xue et al. [166] also observed similar results in leaves of both young and senescing lettuce plant, confirmed that excess Se diminished the antioxidative function, especially decreased activity of GPX and SOD. Moreover, Se bioaccumulation (both SeO_3_^2–^ and SeO_4_^2–^) correlate positively with the GPX activity, where Se-dependent GPX activity was related particularly with the chemical form of Se rather than the Se bioconcentration [116].

Nowak et al. [167] demonstrated that higher Se concentrations (0.15 and 0.45 mM SeO_3_^2–^) caused inhibition in the activity of antioxidant enzymes and provoked stress responses in wheat, where a slow Se concentration (0.05 mM) positively affected the antioxidant defense machinery, Contrary, excess Se-induced prooxidative effect may exert negative impact by reducing guaiacol peroxidase (GPOX) activity. In contrast, a low Se concentration stimulates GPOX activity, positively enhanced antioxidant defense [158,168]. In white clover and ryegrass, high Se concentrations promoted lipid peroxidation, increased shoot Se concentration as well as alters the activities of POD and APX [116,168]. Prooxidative high Se concentration (10 μM) exerted a toxic effect on cucumber also, by overgenerating ROS (O_2_^•–^, H_2_O_2_, HO^•^), resulted in enhanced lipid peroxidation and plasma membrane damage [156]. Oxidative stress caused by Se toxicity decreased respiration intensity, reduced chl content, decreased water balance, and accumulated free proline in common bean [169]. The authors also observed more severe lipid peroxidation compared to H_2_O_2_ damage accompanied by reduced activity of antioxidant enzymes (SOD, CAT, APX, and GR) and content of non-enzymatic antioxidants (AsA and GSH) [126,127,169].

Treatment with SeO_3_^2–^ (50 or 100 μM) was the source of nitrosative modifications in *Arabidopsis*. Distinctive oxidative stress, callose accumulation, pectin accumulation, etc. were well-recognized Se toxicity [26]. Similarly, SeO_3_^2–^ exposure (50 or 100 μM) modified the GSH content, APX, and CAT activities in *Pisum sativum*, where oxidative stress was the outcome of Se-induced nitric oxide-mediated secondary oxidative stress [30]. Selenium exerts nitro-oxidative stress by peroxynitrite overgeneration. Protein tyrosine nitration was also observed in Se affected plants [30]. On the other hand, SeO_4_^2–^ (100 μM) distorted carbohydrates metabolism in wheat along with declined AsA and GSH contents, as well as reduced activities of SOD, APX, and GR. Moreover, PSII and PSI activities were reduced together with distorted redox balance linked to Mn(II)/Mn(III), and semiquinone/quinone ratios under Se toxicity [120]. Mroczek-Zdyrska and Wójcik [158] also observed a similar result in *Vicia faba* plants exposed to SeO_4_^2–^ (6 μM) where severe oxidative stress was justified with higher O_2_^•−^ production, lipid peroxidation, and cell membrane injury, while augmented GPX activity and diminished GPOX activity were evidenced in Se-stressed plants. Moreover, SeO_4_^2–^ treatment (40 and 80 μM) caused oxidation of proteins producing malformed or misfolded in selenoproteins of *Stanleya pinnata* [159]. The cad2-1 mutant of *A. thaliana* under SeO_4_^2–^ (20 μM) stress was characterized by a flawed GSH synthetic pathway, and the root length of these plants was reduced significantly, in contrast to the wild type. In other mutant apr2-1 GSH depletion and ROS accretion were prominent owing to Se toxicity [160]. In turn, Gomes-Junior et al. [170] detected ten SOD isoenzymes with two major Mn-SOD isoenzymes responding more efficiently to 0.05 than 0.5 mM of SeO_3_^2–^. Moreover, an extra glutathione reductase (GR) isoenzyme, which has the potential for oxidative stress in coffee, was induced by SeO_3_^2–^. However, the current challenges will probably be focused on discrimination between Se toxicity induced by oxidative/nitrooxidative stress and non-specific Se-proteins.

## 6. Phytoremediation of Selenium-Contaminated Environments

Phytoremediation, also known as green biotechnology, is an approach to eliminate toxic elements from the contaminated environment using various plant species. Further, toxic elements can effortlessly be removed through plant harvesting or volatilized into their less harmful volatile forms. Phytoremediation is recognized as eco-friendly and cheaper than other methods. It does not affect soil fertility, like some engineering interventions [13,19]. Almost all plants easily uptake Se, and this phenomenon could be implemented both for the removal of Se from the contaminated areas and for the biofortification of plants [33]. Table 3 shows the list of plant species (crops and non-crops) used for Se phytoremediation.

**Figure 6:**
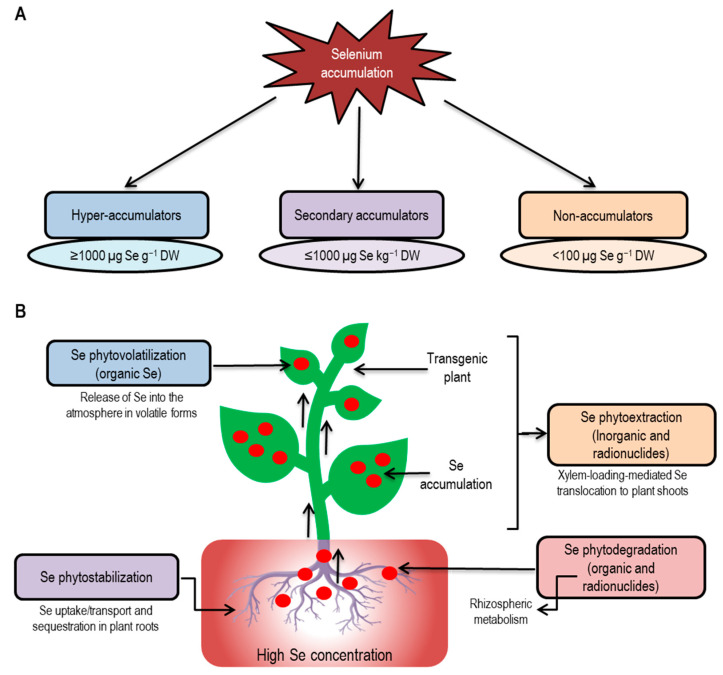
Phytoremediation of Se-polluted environments. (**A**) plants types according to Se accumulation in biomass), (**B**) phytoremediation processes of Se polluted environments. Various phytotechnologies can be used to remediate Se contaminants by accumulating them at large amounts in various parts of plants, especially transgenic plants can provide safe and quick Se phytoremediation to avoid the adverse environmental impact and toxicity to consumers. The main bioavailable form of Se in soils is SeO_4_^2–^. After uptaken up by plants, SeO_4_^2–^ can be accumulated in the root and easily translocated to the shoots. Inorganic SeO_4_^2–^ can be integrated into Se-Cys and other forms of organic Se. A few types of organic Se are volatile and can be released by the plants into the atmosphere as a harmless gas. Selenium accumulation and volatilization may be used to produce Se-biofortified crops and in phytoremediation, respectively.

The choice of plant species for phytoremediation is crucial for the successful remediation of the Se-contaminated environment. Transgenic plants can also be used to enhance phytoremediation capacity. Different phytoremediation approaches, such as phytoextraction, phytovolatilization, and rhizofiltration are widely used for the remediation of Se-contaminated environments (Figure 6).

### 6.1. Selenium Hyperaccumulation

Although Se is beneficial for many of the plants and animals, its essentiality for plants is not recognized yet. There are variations among plant species regarding uptake and accumulation of Se as well as producing volatile Se-compounds to avoid Se toxicity [19,192]. Therefore, according to the capacity to uptake, utilize, and accumulate Se, plants are categorized into three classes [Se hyperaccumulators (accumulate ≥ 1000 µg Se g^−1^ DW), secondary Se accumulators (accumulate 100–1000 μg Se kg^−1^ DW), non-accumulators (contain < 100 μg Se g^−1^ DW). The secondary Se accumulators can grow in seleniferous, and non-seleniferous soils and are termed as the Se-indicators, as their tissue Se content indicates the Se-phytoavailablity [19,148,193]. Nevertheless, there are variations among plants in terms of Se accumulation and tolerance within individual groups, genus, species, subspecies, ecotypes, or even cultivars [148,194,195].

In 1930, a group of researchers led by Orville Beath discovered Se hyperaccumulation and described indicator plant species grown on seleniferous soils. Thereafter, Se hyperaccumulators were identified in 6 families, 14 genera, and 45 taxa of the plant kingdom [148]. Among them, 25 Se hyperaccumulating taxa were reported in the family Fabaceae genus *Astragalus*. Others belong to the Brassicaceae family (species *Stanleya pinnata* and *S. bipinnata*) and the Asteraceae family (genera *Oonopsis*, *Xylorhiza,* and *Symphyotrichum*) [196]. Moreover, *Neptunia amplexicaulis*, a member of the Fabaceae family, is reported to be Se hyperaccumulator when growing on seleniferous soil. Although the Se content in a hyperaccumulator species could be up to 1.5% of their DW, there might be genetic variation among populations and within species [195]).

Previously it was stated that SeO_4_^2−^ is chemically similar to SO_4_^2−^, and can occupy the SO_4_^2−^-transporters (SULTR1;2 and SULTR1;1) to enter the root cells and move in the whole plant [132]. Many factors are responsible for uptake and transport of the SeO_4_^2−^/SO_4_^2−^, for instance, plant species, Se:S ratio in plant organs, and in growing media [19,195,197]. In hyperaccumulators, the expression of SO_4_^2−^ transporters is higher in comparison to secondary accumulators and non-accumulators, which is responsible for elevated Se content in their tissues. Moreover, the overexpression of SO_4_^2−^-transporters in the hyperaccumulators bestow them not only to uptake but also to translocate Se to the aboveground plant organs [197].

Hyperaccumulators have some other traits that enable them to survive and grow successfully on Se rich soils. These species can efficiently convert the inorganic Se into non-protein organic Se and reduce the risk of oxidative stress [198]. They also uptake organic Se forms (Se-Cys, Se-Met, and MeSe-Cys) directly [199], but have the capability of preventing them from incorporation into proteins. Moreover, they can maintain the appropriate amount of Se in the tissue by the enzymatic transformation of MeSe-Cys to volatile dimethyldiselenide [193]. Additionally, they can transform Se-Cys to Se^0^ by the activity of selenocysteine lyase and preventing Se-Cys incorporation into the protein [200]. Furthermore, Se hyperaccumulators may sequestrate organic Se to tolerate Se toxicity. A considerable number of reports suggest that Se is involved in upregulating the antioxidant defense in the hyperaccumulator species, where enzymatic and non-enzymatic antioxidants and phytohormones, i.e., jasmonic acid (JA), salicylic acid (SA), and ethylene (ET), play key roles in Se tolerance [127,201,202].

Mechanisms of Se hyperaccumulation are of great interest to Se researchers. Hyperaccumulators may have evolved independently in different taxonomic families, genera, and species under similar ecological and physiological selection process [171,172,195,200]. Therefore, these traits might be beneficial both for Se phytoremediation and its biofortification.

### 6.2. Phytoextraction

Phytoextraction includes the harvesting of plants for the dismissal of metals/metalloids from contaminated soil; this approach is economical and ecofriendly but not very productive due to less phytoavailability of metals in soils and quite slow [171,203,204].

Various plants grown on Se-contaminated soils are Se-hyperaccumulators, but they show moderate growth, and low biomass production leads to inadequate removal of Se [205]. Due to the high potential of accumulation, some *Brassica* species, like rapeseed and mustard, were recognized for phytoextraction of Se from contaminated areas.

Chelating agents such as EDDS, DTPA, and ethylenediaminetetraacetic acid (EDTA) are considered as a potential weapon to increase the availability of metal/metalloids for increasing the efficiency of phytoextraction. However, the utility of various chelating agents depends on the plant species and the elements to be removed [174,206,207]. On the other hand, chelator-assisted phytoextraction may induce water contamination as they increase the mobilization of toxic ions, ultimate leaching [208,209]. In *Brassica oleracea,* Esringü and Turan [174] reported that the application of EDDS (7.5 mmol kg^−1^) and DTPA (1 mmol kg^−1^) increased Se removal by 12–20 fold from the contaminated soil.

The alteration in physiochemical properties of soils like pH, organic carbon, chelators, and Eh may affect the uptake and phytoaccumulation of Se [210]. However, Bañuelos et al. [171] showed that *Brassica* plants removed approx. 50% and barley approx. 20% of the Se from the soil. Besides, Johnsson [211] claims that 1.4 to 39% increased organic amendments in the plow layer decreased Se level from 1350 to 150 μg kg^−1^ in wheat seeds. Later, Dhillon et al. [212] reported that the addition of chicken manure and sugarcane press might reduce the uptake of Se by 44–97%. On the other hand, Yadav et al. [213] indicated onion as a Se remediator from contaminated soils in a cropping system. Hence, the attempts of phytoextraction for various Se-contaminated soils should be focused on the utilization of Se-enriched plant biomass, unescorted by the chelating agents.

### 6.3. Phytovolatilization

Plants can convert a toxic form of Se into less toxic compounds, e.g., volatile organic seleno-compounds. The process in which plants take up the pollutants from soil and release them in a volatile form is regarded as phytovolatilization [214]. The release of volatile organic elements from stems and leaves is known as direct phytovolatilization, while the increase in volatile contaminant flux from the contaminated soils by the root activity is known as indirect phytovolatilization [214]. The key benefit of this approach is, it can eliminate the contaminants without plant harvesting and/or biomass utilization.

Beath et al. [178] first reported Se volatilization by Se-hyperaccumulator (*A. bisulcatus*). Afterward, Evans et al. [215] found that the basic evaporative Se form released by Se-hyperactive plants was DMe-DSe. While non-accumulator *B. oleracea* released DMe-Se [216]. Dumont et al. [67] showed that these volatilized forms are nearly 600-fold less harmful compared to inorganic Se. Among various plant species, cabbage and *A. bisulcatus* volatilize a higher rate of Se, followed by alfalfa and tomato [37]. Later, Bañuelos et al. [217] and Terry and Zayed [218] reported that plants of the Brassicaceae family (cabbage and broccoli) have higher volatilization capability. The slow growth and low biomass production of Se-hyperaccumulators limit their potential for phytoremediation [172]. Hence, the combined effect of phytovolatilization and phytoextraction can increase the phytoremediation efficiency by two to three-fold.

Selenium volatilization efficiency is based upon a variety of factors, such as plant type, Se form, the configuration of the microbial group, type of macrophytes, temperature, the existence of other elements in the growing medium, microorganisms in the rhizosphere, and many other physiochemical parameters [38,218,219]. Selenium volatilization increases with temperature; higher temperature also improves the metabolic activities of plants [38,219]. Therefore, it is still required to explore the effects of these factors on phytovolatilization efficiency in field experiments.

### 6.4. Rhizofiltration

Rhizofiltration is a sub-technique of phytoremediation, which employs a plant root system to absorb contaminants, mainly toxic metals, from solution surrounding the rhizosphere, groundwater, surface water, and wastewater [220]. Suitability of various aquatic plants for Se rhizofiltration have been studied through short-term experiments in aqueous solutions including *Myriophyllum brasiliense*, *Potamogeton crispus*, *Juncus xiphioides*, *Typha latifolia*, *Ruppia maritima*, *Scirpus robustus*, and *Hydrilla verticillate* [220,221,222,223], and long-term experiments in constructed wetlands [224,225].

Cattail (*Typha angustifolia*) grown in wetland conditions has been reported effective in the removal of Se applied as SeO_3_^2–^ and SeO_4_^2–^ by 89% and 46%, respectively [38], while musk grass removed about 70–75% of supplemented Se from the aqueous environment [179,180]. Duckweed (*Lemna minor*), known for the natural capacity to accumulate Se, removed 55–99% of supplemented Se [222,226]. In turn, soft rush (*Juncus effusus* L.) can be used for Se rhizofiltration due to its availability in wetlands compared to cattail. Miranda et al. [222] demonstrated that the biomass of many aquatic plants possesses a considerable potential for the production of biofuel. Hence, the dual advantage of aquatic plants for polluted water management and the generation of renewable fuels and petrochemicals came up with an eco-friendly and cheap way for the remediation of Se-contaminated water.

### 6.5. Genetic Engineering for Se Phytoremediation

Genetic engineering is the modern tool used nowadays to enhance plant abiotic stress tolerance and in phytotechnologies (phytoremediation and biofortification). Recent advancements in omics approaches allowed altering plants at the molecular level resulted in efficient phytoremediation of Se [227]. The basics are to modify gene expression to target different routes and pathways for phytoremediation in non-accumulators, or secondary accumulators or to transfer the traits into a slow-growing hyperaccumulator (Table 4) [175]. Several researchers have successfully adopted the transgenics to enhance the Se-tolerance, Se-accumulation, as well as Se-volatilization using the traits from the Se-hyperaccumulators.

After entering in the root cell, SeO_4_^2−^ is first reduced to SeO_3_^2−^ with the ATP sulfurylase (APS), which is the initial step for the assimilation of SeO_4_^2−^ to organic Se. Therefore, an attempt was made to overexpress APS from *A. thaliana* in *B. juncea*. Consequently, the transgenic plants showed two to three-fold increased Se accumulation compared to the unaltered plants, but this transformation had no impact on the Se volatilization rate. In the Se metabolism pathway, the cystathionine-γ-synthase (CgS) enzyme is responsible for the conversion of Se-Cys to Se-Met, which is further converted to volatile DMe-Se. Therefore, *A. thaliana* CgS gene overexpression in *B. juncea* resulted in two to three-fold increased volatilization efficiency in comparison with untransformed plants [228].

As mentioned before, plants can take up Se-amino acids and incorporate them into the proteins leading to Se toxicity. To prevent Se-Cys incorporation to proteins, the enzyme selenocysteine methyltransferase (SMT) converts the Se-Cys to MeSe-Cys. Considering this, an attempt to overexpress the SMT gene from *A. bisulcatus* in both *B. juncea* and *A. thaliana* resulted in upregulated Se accumulation and Se tolerance, as well as increased Se-volatilization [231,232]. However, these plants were more efficient in Se volatilization when exposed to SeO_3_^2−^ than SeO_4_^2−^. Therefore, an attempt to overexpress two enzymes (APS and SMT) showed approximately nine-fold higher Se-accumulation, where the majority of the Se was in MeSe-Cys form and 8-fold higher compared with wild plants [234]. Notably, Se tolerance, both single and double transgenics were the same. In turn, overexpression of Se-Cys lyase, an enzyme converting Se-Cys to Se^0^, in *A. thaliana* and *B. juncea* resulted in enhanced Se accumulation in comparison with the wild type [229,230].

## 7. Conclusions and Outlook

In this review, we discussed the causes of Se phytotoxicity, mechanisms of Se-induced cell damage, as well as Se biogeochemistry and phytoremediation features. A high amount of Se exerts several negative and harmful effects on the plant due to oxidative stress, altered and malformed protein structure, disrupted enzymatic function, interrupted biosynthesis and metabolism of carbohydrates, proteins, and other metabolites, distorted chloroplast, and mitochondrial ultrastructure and functioning. These negative effects considerably reduce plant growth, development, and overall production. Selenium has a very narrow gap between its adequacy and toxicity. Consequently, both Se-inadequacy and toxicity are widespread globally and overlap with soils that are low and rich in Se, respectively. Although some recent publications revealed both positive and negative effects of Se, there are still various aspects of Se biological action that required to be revealed, e.g., the essentiality of Se for plants and selection of plants show enhanced growth under Se exposure. Additionally, the effective concentrations of Se inducing a positive or negative effect on plant growth, development, and ecophysiology should be determined. Moreover, the actual mechanisms causing these effects should be revealed. It is also vital to explain the connection between Se and S biogeochemistry, which affects Se and S uptake in natural conditions. Moreover, plant tolerance to Se, their remediation potential, and Se detoxification mechanisms need to be enhanced for efficient phytoremediation of Se polluted areas.

Promising plant species for phytoremediation, phytoextraction, phytovolatilization, or rhizofiltration to reduce the Se concentration in polluted soils should be identified. Additionally, some exciting features of Se hyperaccumulators are still required to be revealed. For example, why and how these unique plant species vary in absorbing and accumulating Se, by which mechanisms plants trigger Se hyperaccumulation, the advantages and disadvantages of Se accumulation in plants, etc. Notably, the development of genetically engineered transgenic *Brassica* plants has a great potential to remove Se at a higher rate, which will probably help to remediate the Se from the polluted environment within a short period.

Therefore, to gain more insight into Se tolerance and toxicity, the results from several genomic, biochemical and genetic engineering experiments and the overexpression of the key Se and S accumulation pathways should be compared. Further, the state-of-the-art omics approaches, mainly transcriptomics, metabolomics, and proteomics, can help to identify the key genes, metabolites, proteins, and regulators encoding actual transporters of selenocompounds into and within hyperaccumulator plants; and the metabolic pathways responsible for the Se translocation. Afterward, the overexpression of such key genes would help to develop the Se hyperaccumulators with high biomass production for more effective remediation of Se polluted areas. Additionally, the engineered Se-associated metabolic pathways can provide novel ideas into the existing knowledge and aid to further discover the Se translocation mechanisms for future investigations.

## Figures and Tables

**Table 1:** Toxic effects of Se on the growth and physiological processes in plants.

Plant Species	Form and Dose of Se	Negative Impact on Growth and Physiology	Reference
*Arabidopsis thaliana*	SeO_3_^2–^; 50 or 100 μM	Se-induced secondary nitrooxidative stress. Decreased root growth and biomass (FW and DW). Reduced cell viability. Modified cell wall structure by modifying the pectin and callose. Decreased stomatal density and impaired stomatal regulations sensitive varieties were affected more than the tolerant.	[26]
*Raphanus sativus*, *Helianthus annuus*, *Medicago sativa* *Beta vulgaris* var. *cicla*	SeO_3_^2–^; 5 or 10 mg Se L^–1^	Growth inhibition.	[115]
*Pisum sativum* cv. Petit Provençal	SeO_3_^2–^; 50 or 100 μM	Altered vegetative and reproductive development. Shoot and root length and FW decreased. Chl *a*, chl *b*, chl *a*/*b*, total chl, total carotenoids content decreased.	[30]
*Cucumis sativus* cv. Polan F1	SeO_4_^2–^; 80 µM SeO_3_^2–^; 20 µM	Decreased shoot root growth, biomass and leaf area. Impaired nutrient content. Reduced photosynthetic pigments accumulation and chl fluorescence. Increased lipid peroxidation.	[117]
*Oryza sativa*	SeO_3_^2–^; 100 g Se ha^−1^	Increased Se content in root and shoot. Reduced photosynthesis and transpiration rate, and intercellular [CO_2_]. Impaired PSII quantum yield and diminished potential photosynthetic capacity. Reduced grain yield.	[118]
*Lactuca sativa* var. *capitata* cv. Justyna	SeO_4_^2–^; 20 µM SeO_3_^2–^; 15 µM	High accumulation of Se and S. Decreased biomass and leaf area. Reduced concentrations of photosynthetic pigments. Increased lipid peroxidation and H_2_O_2_ accumulation.	[119]
*Triticum aestivum*	SeO_4_^2–^; 100 μM	Reduction of PSII and PSI activities.	[120]
*A. thaliana*	SeO_4_^2–^; 20 or 40 μM	Root growth inhibition. Loss of root apex cell viability and malformed root architecture. Reduction of primary root growth, an increase of lateral root growth. Decreased meristem cell activities. Hormonal imbalance.	[121]
*Spinacia oleracea* cv. Missouri	SeO_3_^2–^; 6 mg L^−1^	Increased Se accumulation. Decreased growth parameters, e.g., shoot and root length, and FW and DW. Increased Na and Ca content, but decreased K content.	[122]
*Ulva* sp.	SeO_4_^2–^; 100 μM	Decreased level of chl and carotenoids.	[123]
*Brassica juncea*	SeO_4_^2–^; 80 μM	Augmented Se and S concentration in different floral parts. Increased floral Se accumulation and impaired pollen germination.	[124]
*Lactuca sativa*	SeO_3_^2–^ and SeO_4_^2–^; 20 µM	Increased shoot Se concentration. Decreased P, S, Mg, Mn, and Fe concentrations. A slight reduction in shoot DW and yield.	[125]
*Hordeum vulgare*	SeO_4_^2–^; 2, 4, 8, or 16 ppm	Decreased plant height. Reduced chl concentrations.	[126]
*Stanleya albescens*	SeO_4_^2–^, 20 μM	Reduced growth. Chlorosis and impaired photosynthesis. Accumulation of the free amino acid selenocystathionine, a carbon-Se-carbon compounds (presumably selenocystathionine) together with some selenocysteine and selenate.	[127]
*Pteris vittata*	SeO_4_^2–^; 50 and 100 mg kg^−1^ in soil.	Suppressed uptake of Mg, K, P, Fe, Cu, and Zn.	[128]
*Lactuca sativa* var. *capitata*	SeO_3_^2–^; 20 μM	Decreased productivity. Declined macronutrients accumulation in leaves.	[129]
*Zea mays*	SeO_3_^2–^; 50 and 100 µmol L^−1^	Decreased DW accumulation. Root tolerance index severely decreased.	[130]
*Z. mays*	SeO_4_^2–^ or selenomethionine (C_5_H_11_NO_2_Se); 100 µM	High Se accumulation in root and shoot. Reduction in root and shoot FW. Altered anthocyanin level. Reduced chl level.	[131]
*Chlamydomonas reinhardtii*	SeO_3_^2–^ and SeO_4_^2–^; 4.5 ± 0.2 µM	Photosynthesis disorders. Ultrastructural damage. Inhibition and interruption of the photosynthetic electron transport chain. Growth inhibition.	[111]

**Table 2:** Evidence for Se-induced oxidative stress in plants.

Plant Species	Form and Concentration of Se	Indicators of Oxidative Stress and Changes in Antioxidant Enzymes Activities under Se Exposure	Reference
*Arabidopsis thaliana*	SeO_3_^2–^; 50 or 100 μM	Distinct oxidative stress. Nitrosative modifications. Callose accumulation. Pectin accumulation.	[26]
*Pisum sativum*	SeO_3_^2–^; 50 or 100 μM	Increased H_2_O_2_ concentration in leaves and roots. Increased content of thiobarbituric acid reactive substances (TBARS). Altered GSH content, APX and CAT activities. Increased nitric oxide level in shoot and root. Nitric oxide-induced nitrooxidative stress by increasing peroxynitrite formation, as well as tyrosine nitration.	[30]
*Brassica rapa*	SeO_3_^2–^; 0.03–0.46 mM	Increased endogenous total ROS, O_2_^•−^, and enhanced lipid peroxidation. Loss of plasma membrane integrity in the roots.	[157]
*Triticum aestivum*	SeO_4_^2–^; 100 μM	Altered carbohydrates (soluble and starch) level. AsA and GSH contents were modified. Suppressed activities of SOD, APX, and GR. Higher generation of ROS. Augmented lipid peroxidation. Repressed PSII and PSI system activities. Modified redox status connected with Mn(II)/Mn(III), and semiquinone/quinone ratios.	[120]
*A. thaliana*	SeO_4_^2–^; 20 and 40 μM	Decreased NO content. Increased H_2_O_2_ content. Reduced cell viability.	[121]
*Vicia faba*	SeO_4_^2–^; 6 μM	Elevated lipid peroxidation and total -SH (T-SH) content. Increased GPX activity. Decreased guaiacol peroxidase (GPOX) activity. Increased O_2_^•−^ production in the roots. Cell membrane injury and reduced cell viability.	[158]
*Stanleya pinnata*	SeO_4_^2–^; 40 and 80 μM	Oxidized proteins. Malformed or misfolded selenoproteins.	[159]
*Ulva* sp.	SeO_4_^2–^; 100 μM	Increased accumulation of H_2_O_2_. The activity of antioxidant enzymes such as SOD, CAT increased. Antioxidant metabolites including phenols, flavonoids, carotenoids, and gallic acid increased.	[123]
*A. thaliana*	SeO_4_^2–^; 20 μM	The cad2-1 mutant was recognized with a flawed GSH synthetic pathway that showed decreased root length, in contrast to the wild type. In the apr2-1 mutant, GSH depletion and ROS accretion were prominent.	[160]
*Hordeum vulgare*	SeO_4_^2–^; 4, 8 and 16 ppm	Increased membrane lipid peroxidation. Higher proline accumulation. Stimulated CAT, APX, GR, and glutathione-*S*-transferase (GST) activities.	[126]
*Stanleya albescens*	SeO_4_^2–^; 20 μM	Increased O_2_^•−^ and H_2_O_2_ levels. Reduced AsA and GSH content. Declined radical-scavenging capacity.	[127]
*A. thaliana*	SeO_4_^2–^; 50 mM	Decreased GSH level.	[161]

**Table 3:** List of selected plant species used for Se phytoremediation.

Plant Species	Family	References
*Brassica oleracea* var. *capitata*, *B. oleracea* var. *italica*, *B. oleracea* var. *botrytis*, *B. juncea*, *B. napus*, *Stanleya pinnata*	Brassicaceae	[35,171,172,173,174]
*Gaillardia aristata* and *Calendula officinalis*	Asteraceae	[175,176,177]
*Astragalus bisulcatus*	Fabaceae	[171,178]
*Arundo donax*, *Triticum aestivum*, and *Oryza sativa*	Poaceae	[36,153,179]
*Eichchornia crassipes*	Pontederiaceae	[180]
*Populus* spp.	Salicaceae	[181]
*Lemnoideae* spp.	Lemnaceae	[182,183]
*Hippuris vulgaris* L.	Plantaginaceae	[184]
*Typha latifolia*	Typhaceae	[185]
*Ipomoea purpurea*	Convolvulaceae	[186]
*Azolla caroliniana*	Salviniaceae	[187]
*Pteris vittata*	Pteridaceae	[188]
*Juncus xiphioides*	Juncaceae	[189]
*Bolboschoenus maritimus*	Cyperaceae	[189]
*Chara* spp.	Characeae	[38,39]
*Corchorus capsularis*	Malvaceae	[190]
*Eucalyptus globulus*	Myrtaceae	[191]

**Table 4:** Transgenic plants and the candidate genes for the targeted Se-phytoremediation.

Transgenic Species	Gene Transferred	Effects	Reference
*Brassica juncea*	Cystathionine-γ-synthase (CgS)	Increased Se volatilization	[228]
*A. thaliana*	Selenocysteine lyase (SL)	Enhanced Se accumulation	[229]
*B. juncea*	SL	Enhanced Se accumulation	[230]
*A. thaliana*	Selenocysteine methyltransferase (SMT)	Enhanced Se accumulation and volatilization	[231]
*B. juncea*	SMT	Enhanced Se accumulation and tolerance	[232]
*B. juncea*	APS	Three-fold increased Se accumulation in leaves	[233]
*B. juncea*	γ Glutamyl-cysteine synthetase (ECS)	Improved Se accumulation	[233]
*B. juncea*	APS×SMT	Increased Se accumulation under both SeO_4_^2−^ and SeO_3_^2−^ exposure	[217]
*B. juncea*	SL×SMT	Enhanced Se accumulation	[217]
